# Photodynamic Therapy and Central Serous Chorioretinopathy

**Published:** 2012

**Authors:** Lina Siaudvytyte, Vaida Diliene, Goda Miniauskiene, Vilma Jurate Balciuniene

**Affiliations:** Eye Clinic, Lithuanian University of Health Sciences, Kaunas, Lithuania

**Keywords:** Central serous chorioretinopathy, Photodynamic therapy, Pathophysiology

## Abstract

Central serous chorioretinopathy is a common acquired maculopathy. Multiple studies showed that photodynamic therapy is useful treatment for acute and chronic central serous chorioretinopathy. The exact mechanism of photodynamic therapy in treating central serous chorioretinopathy is not clear, but it is thought to be caused by short-term choriocapillaris hypoperfusion and long-term choroidal vascular remodeling, leading to a reduction in choroidal congestion, vascular hyperpermeability and extravascular leakage. Furthermore, photodynamic therapy seems to be an effective means of improving or stabilizing visual acuity in patients with central serous chorioretinopathy.

## INTRODUCTION

Central serous chorioretinopathy (CSC) is characterized by a serous detachment of the neurosensory retina in the macular region, occasionally associated with detachment of the retinal pigment epithelium (RPE). The most surprising aspect of the disease is the relative preservation of retinal function regardless prolonged separation from the RPE. Males are mostly affected to have this condition and the average age is between 20 and 50 years. The usual presenting symptoms are significant loss of visual acuity and development of permanent visual loss. Visual impairment is secondary to persistent serous detachments of the neurosensory retina leading to cystoid edema of the retina and diffuses decompensation of the RPE [1]. The photoreceptors might have a critical role in this process, because they are separated from their source of nutrients when the retina is detached [2]. Some patients, particularly older adults, can develop choroidal neovascularization, which leads to severe visual loss [3].

The pathogenesis of CSC is still not completely understood. However, it is well known that the subneural retinal fluid originates from the choroid. At first it was believed that fluid from the choroid drain away into subretinal space through defects in tight junctions between the RPE cells due to breakdown of the blood-retinal barrier. However, this theory does not explain the beneficial effect of laser photocoagulation which consequences in permanent RPE barrier breakdown. Another theory suggested that lost of normal RPE cells polarity acts as a trigger for fluid pumping from the choroid to the retina, causing a neurosensory detachment [4]. This theory was failed after increased using of indocyanine green angiography (ICGA) which reveals multifocal areas of choroidal vascular hyperpermeability in CSC, which leads to mechanical disruption of RPE barrier with subsequent accumulation of subretinal fluid, supporting the theory that the underlying pathophysiology is at the choroidal level [5,6]. No new vessels are usually present in CSC, but the defect seems to affect choroidal vessels. Any therapy that decreases the excess of choroidal permeability may be potentially helpful in CSC cases [7].

Therefore recent studies examining the pathogenesis of CSC support the hypothesis that RPE decompensation may be a result of underlying choroidal vasculature hyperpermeability [8-11]. Some authors reported that choroidal vascular hyperpermeability was seen in most symptomatic eyes with CSC [4,6]. Prunte et al. showed delayed choroidal capillary lobular filling in areas of hyperpermeability and proposed that localized capillary and venous congestion in the affected lobules impaired the circulation, produced ischemia, and allowed increased choroidal exudation and a focally hyperpermeable choroid [12]. Increased choroidal permeability along with local high perfusion and increased hydrostatic pressure allows profusion choroidal fluid to accumulate and produces a RPE detachment. As the detachment grows, the target junctions between RPE cells are broken, and a focal defect of the blood-retinal barrier develops, later resulting in neural retinal detachment [13-14]. Some investigators revealed that subfoveal choroid in the eyes with CSC, even in the fellow eyes are thicker than that in normal eyes because of choroidal vascular hyperpermeability [9,15]. They used optical coherence tomography (OCT) to evaluate choroidal hyperpermeability by measuring choroidal thickness. Interestingly, recent studies reveal that corticosteroids can influence the production of the nitric oxide, prostaglandins and free radicals within the choroidal circulation. All three substances participate in the autoregulation of blood flow within the choroid [16]. 

The treatment of central serous chorioretinopathy has not been well-established. Different therapeutic approaches have been tried to manage this condition, including beta-blockers, acetazolamide, vitamins and non-steroidal anti-inflammatory drugs, but none of these had explicit benefits [17]. In past decades, argon laser photocoagulation of extrafoveal leakage points was the standard of CSC treatment [18,19]. It is the only therapy proved beneficial by large clinical trials. Laser treatment induces a local inflammatory reaction on RPE, thus decreasing RPE leakage while choroidal hyperpermeability remain unchanged [20]. The evidence of long follow-up studies shows a reduction of serous detachment with lack of improvement in final visual acuity or a reduction in the incidence of recurrences [21-23]. Laser photocoagulation cannot be performed in the foveal avascular zone. Laser therapy may result adverse effects such as secondary choroidal neovascularization or central scotomas [24-26].

Another treatment option is photodynamic therapy (PDT). PDT originally was intended to cause regression of choroidal neovascularization (CNV) secondary to age related macular degeneration and recently is used for neovascular age-related macular degeneration, pathologic myopia and ocular histoplasmosis caused CNV treatment. The exact mechanism of PDT on CSC is not well-known. It has been suggested that PDT may induce choriocapillaris damage and vascular remodeling thus decreasing choroidal hyperpermeability [12,27-32]. Maruko et al. using enhanced depth imaging OCT, reported reduced choroidal thickness 1 month after PDT treatment in chronic CSC patients [20]. These findings are compatible with ICGA showing a transitory hypoperfusion [11]. Another authors hypothesized that PDT acts by both decreasing choroidal hyperpermeability and tightening the blood retinal barrier at the level of the RPE resulting in resolution of subretinal fluid [1].

Patients with chronic forms of CSC may benefit from a decreased choroidal vascular permeability. Some authors suggest that verteporfin may show a high affinity for RPE [1,33]. Verteporfin is a benzoporphyrin derivate which is used as a photo sensitizer for PDT to eliminate the abnormal blood vessels in the eye. It is known that the primary effect of PDT seems to be damage of the choriocapillaris endothelium, swelling, fragmentation, detachment from its basement membrane and degeneration [31]. Another possible explanation for the positive effects of this therapy concerns the inflammatory reaction precipitated by PDT. Verteporfin may be deposited within the serous fluid under the macula and its activation may release free radicals and pro-inflammatory factors that induce a permanent adhesion between the neurosensory retina and RPE. This mechanism may explain the occurrence of inflammatory changes in the RPE [7]. Otherwise the vascular endothelial damage known to be the major hallmark of photodynamic tissue effects is induced by direct interaction of singlet oxygen with the lipids of the endothelial cytoplasmic membranes. Recanalization of the choriocapillaris begins to occur within a short interval after doses of therapy. Maintenance of structural integrity histologically of the overlying photoreceptors seems to be the result of limited hypoxia or thermally enhanced phototoxic damage [31]. Histologic studies on animal models and humans have shown that PDT induces the regression of subretinal newly formed vessesls as well as obliteration of the vessels of the inner choriocapillaris [32]. 

 The standard regime for using PDT is to give patient intravenous verteporfin at a dose of 6 mg/m2 over 10 minutes. Then, 5 minutes later, diode laser at a wavelength of 600-689 nm and energy of 50 mJ/cm2 over 83 seconds is directed to the target lesion of the eye. Possible ocular side effects include RPE atrophy and rips, secondary choroidal neovascularization, and ischemia of choriocapillaris. To minimize adverse events, research has targeted half-dose half fluence and minimal-fluence PDT [35]. Half-dose or low-fluence PDT with verteporfin is effective in inducing reabsorption of subretinal or intraretinal fluid with some beneficial visual outcomes in the majority of patients with CSC [33,45].

PDT is not completely harmless to ocular structures [20,30-32,36-40]. Choriocapillaris thrombosis and choroidal perfusion and permeability changes have been demonstrated. These reports show no early neural retina or RPE changes with standard verteporfin doses [30,31]. Some studies showed that standard dose PDT might be associated with choriocapillaris hypoperfusion that may result in decreased vision [41]. In recent years, several different studies have supported the good results of PDT with standard doses of verteporfin to treat chronic CSC [7,33,35-47]. However, these studies were performed with a small number of patients and short follow-up. In order to avoid PDT related complications half-dose or low-fluence PDT has been suggested by different authors. Higher selectivity of the choriocapillaris was achieved with a lower fluence PDT, while higher fluence emission resulted in closure of the deeper choroidal vessels and focal alterations in the RPE.

PDT has shown better results on visual acuity and anatomical outcome compared with photocoagulation in chronic forms of CSC, with fewer complications [1,7,45-47,49-53]. Changes in the average neural retina thickness of eyes treated by PDT could be supported by the long-standing effects of vascular remodeling in the underlying choroid [20,30]. Photodynamic therapy with verteporfin might induce temporary choriocapillaris occlusion and endothelial changes, this might reduce the vascular permeability and decrease fluid passage toward the retina [45,49]. Moreover, RPE cells damaged by light-activated verteporfin might be replaced by new ones possible recovery from the metabolic impairment at the RPE level [34,49].

## HYPOTHESIS

Clinical and experimental evidence indicates that besides closing the neovascular membrane this treatment also produces ischemia of the underlying choriocapillaris, induced by direct action on the choriocapillaris endothelium with choriocapillaris occlusion and resulting in hypoperfusion of the choriocapillaris in the short term and remodeling of choroidal vascular over time. This effect of PDT on the choroid could be used to reduce choroidal congestion and vascular hyperpermeability, which is an important factor in CSC pathogenesis. 

## CONCLUSION

PDT seems to be an effective therapy of improving or stabilizing visual acuity in patients with central serous chorioretinopathy. However, more studies are needed to manifest the benefits, efficacy and long-term safety of PDT in the treatment of CSC..

## DISCLOSURE

The authors report no conflicts of interest in this work.
